# COVID-19 in Rural Ontario Communities: Exploring Women’s Mental Health During a Pandemic

**DOI:** 10.3390/ijerph22060937

**Published:** 2025-06-13

**Authors:** Amanda Norton, Laura Rosella, Matthew Adams, Leith Deacon

**Affiliations:** 1 Department of Geography and Planning, University of Toronto, Room 5047, Sydney Smith Hall, 100 St. George Street, Toronto, ON M5S 3G3, Canada; md.adams@utoronto.ca; 2 Division of Epidemiology, Dalla Lana School of Public Health, University of Toronto, 6th Floor, Health Sciences Building, 155 College Street, Toronto, ON M5T 3M7, Canada; laura.rosella@utoronto.ca; 3 Department of Geography, Geomatics, and Environment, University of Toronto Mississauga, DV3261, 3359 Mississauga Road, Mississauga, ON L5L 1C6, Canada; 4 Department of Rural Planning and Development, School of Environmental Design and Rural Development, Guelph University, Room 122, Landscape Architecture, 124 Reynolds Walk, Guelph, ON N1G 2W1, Canada; leith.deacon@uoguelph.ca

**Keywords:** mental health, access to care, geography, health disparities

## Abstract

Purpose: Socio-demographic inequities in mental health were magnified by COVID-19, with women experiencing greater household burden with less support in Canada and globally. While some health patterns during COVID-19 have been observed globally, there is a research gap in rural mental health during COVID-19 in Canada. We hypothesize there is a disparity in mental health decline during COVID-19 between men and women. Methods: In rural Ontario, mental health was measured through a survey of approximately 18,000 individuals living in seven counties. In 2021, survey respondents were asked to rate their mental health prior to and during COVID-19. Women reported poorer mental health during COVID-19 in comparison to men when tested via chi-squared tests, odds ratios, and percentage change. Responses to survey questions regarding social, financial, and mental health support were then evaluated. Findings: We found significant disparities in mental health ratings before and during COVID-19 between men and women. Women reported poorer mental health, increased substance use, and increased worry about social, financial, and community stressors. Respondents who self-identified as a woman were associated with poorer mental health outcomes. Conclusions: Interventions should be specific to geographic communities as well as individual needs (e.g., additional financial and childcare support). Rural communities need to be considered as independent geographies rather than as one geography (i.e., urban vs. rural).

## 1. Introduction

Mental health during COVID-19 declined in populations in Canada; incidence of depression, anxiety [1], and suicidal ideation increased [2], demonstrating a need for improved access to care. Populations living in rural areas, in comparison to urban populations, had a higher risk burden as mental health resources were strained [3]. Urban areas had high COVID-19 case counts and accounted for most cases in Ontario [4]; however, Ontario’s rural areas suffered [5]. Gender inequities in the division of household work were magnified during COVID-19, with women facing increases in career disruptions (as it is more difficult to work from home with children), household work (added cooking and cleaning), and childcare in comparison to men (as outside-of-home childcare reduced) [6,7]. In addition to increased workload, quarantine caused reported increases in intimate partner violence [8]. Mental health in rural areas of Ontario [5] and across Canada declined as a result of social isolation and reduced social support [9].

During COVID-19, many recommended interventions such as food delivery services (e.g., DoorDash, UberEats, Skip), remote work, and telehealth were developed with urban communities in mind; rural environments often are well beyond delivery boundaries and, at times, are beyond internet connection [10]. Rural environments need to be considered independently when developing health interventions. In addition, there is variation within rural communities, meaning that some may have had more resilience in the context of COVID-19 than others.

The relationship between rural mental health and COVID-19 has not yet been explored in the context of gender in Ontario. Rural populations faced unique challenges during COVID-19. This analysis will investigate how COVID-19 impacted perceived mental health and if that perception differed between men and women living in seven rural Ontario counties. Declining mental health in women as a result of COVID-19 has been studied provincially [11,12] and nationally. Few studies, however, have examined the intersection of gender, rurality, and mental health across and within Ontario [13].

### 1.1. Rural Ontario and COVID-19

Ontario administrative health data indicated that children and adolescents living in Ontario had lower than expected (pre-pandemic) uptake of mental health visits in the months immediately following the onset of the COVID-19 pandemic [14]. Five months after the pandemic, there were higher than expected rates of mental health visits for children living in urban neighborhoods, and six months after, there were higher than expected rates for children living in rural neighborhoods [14]. The pre-pandemic expected rate for mental health visits was lower than in urban areas, and while insignificant, there was a slightly lower (0.1) observed difference in rural mental health visit uptake [14]. External stressors which impact mental health were exacerbated by the pandemic. In Ontario, family courts were forced to go virtual, further delaying critical justice processes and increasing self-representation [15]. Those living in rural communities reported heightened concerns around access to the courts, new technology, and internet connectivity [15]. External stressors have been linked to family cohesion which in turn has been linked directly to poorer mental health (increased anxiety) [16].

In a study of ten Ontario workers, lack of regulatory support, structural support (i.e., policy or management), and gender biases were identified as barriers to reception of adequate protections from COVID-19 [17]. Female workers reported distress as a result of their caretaking responsibilities at home and work, fearing they were putting those in their care at risk [17]. Cleaning protocols for COVID-19 in healthcare were reported to be inconsistent, sometimes changing daily, increasing lack of confidence in protective measures [17]. In rural Ontario, between May 2021 and August 2021 physicians self-reported increases in changes in depression and anxiety and decline in well-being due to increased infection risk, lack of resources, and care demands [18]. Burnout (defined as chronic workplace stress) [19] in healthcare providers has been associated with poorer healthcare delivery [18].

Ontario studies have explored substance use and rural health outcomes during COVID-19. There appeared to be an increase in disparities of alcohol-related hospitalizations (all-cause ED visits) between urban and rural areas, with rural areas having a higher rate of admissions [20]. During the pandemic, there was a decline for both men and women in hospital admissions due to alcohol use, possibly signaling disinclination to utilize healthcare [20]. Healthcare utilization did not decline in rural areas as much as urban areas, indicating healthcare access may not have been a driving force of alcohol-related ED visits; the unique characteristics of rural communities may have led to increased substance use [20].

Rural Ontarians may have suffered from COVID-19′s economic impacts. Policies for controlling COVID-19 were developed for urban regions; lockdowns were implemented in rural areas, despite having lower COVID-19 cases. Lockdowns impacted small businesses and local economies, straining the gap between individuals shopping locally versus shopping at big box stores [21]. To account for inequities between urban and rural environments, it has been suggested that interventions become more geographically specific [22].

### 1.2. Gender and Rural Environments

A 2023 Canadian report on the differences between men and women’s views on social and democratic values in rural environments showed that only 73% of men and 83% of women living in rural environments agreed with gender equality [23]. In comparison, 78% of men and 85% of women living in urban environments supported gender equity [23]. These findings may demonstrate that there is less emphasis on gender equity in rural Canadian environments. Men are an important part of achieving gender equity as they play a role in creating a hospitable work place; further, men’s gender roles may be damaging to their own mental health, creating a cycle [24]. A disproportionate lack of men’s support in gender equity directly influences women’s mental health, possibly causing health disparities between urban and rural environments [25]. A study of senior women living in rural Ontario revealed that the biggest mental health issues women faced were loneliness and negative self-worth, with inadequate resources and devaluing of gender being among the factors contributing to poorer mental health [26]. Women in this study reported feeling ignored in their own communities; however, there was little discussion on why they felt ignored [26]. A lack of importance and sense of belonging in community may influence perceived mental health among women [27].

### 1.3. International Rural Geographies and COVID-19

Internationally, there are distinctive social and structural dynamics in rural communities. In the United States, at one point, rural communities were the epicenter of COVID-19, reflecting a need for an increase in healthcare resources [28]. Qualitative research conducted in Melbourne, Australia reflects that specifically females in rural areas needed more resources during COVID-19 [29]. In Bcharra, Lebanon, the rurality of the community is what strengthened COVID-19 response, as the relationships community leaders developed were leveraged for participation in pandemic response [30]. Each citizen of Bcharra participated in the pandemic response. The pandemic response framework was formed immediately and within the context of the area [30]. Techniques within reach of smaller communities included contact tracing and concise protocol development (as rural areas had a delayed onset of cases) [30].

In Ireland, rural farmers described the extreme isolation experienced by their communities, expressing their concern for farmers that lived alone or were older [31]. The social centers (church, grocery shopping) of these communities, which were already isolated, were eliminated, with some farmers even discussing that it was not feasible or natural to pick up the phone to chat with neighbors [31]. In contrast, parents living on farms were grateful for the space and tasks available to occupy their children [31]. In a study set in rural South India, many people migrated to rural communities during the pandemic; many reported experiencing financial hardship, poorer mental health, and disruptions in healthcare access [32].

### 1.4. Gender and COVID-19

Gender disparities during COVID-19 were a consistent theme. Trouble connecting, burden of care, and financial struggles were among the themes impacting workers at intimate partner violence (IPV) centres during COVID-19 [33]. Management and staff reported experiencing additional burdens at home as a result of school and daycare closures [33]. The need for IPV support during COVID-19 increased, leading to burnout of IPV service providers [33]. Many women’s careers during COVID-19 suffered because of increased household burdens. Canadian mothers reported approximately 30 additional hours of childcare per week. Women in STEM (science, technology, engineering, and mathematics) mostly worked from home (~70%), and 22% reported spending more than 3 h per day on childcare (in comparison to men, who reported 12%) [34]. Women working in academia reported significant gender-based challenges (~20%), in comparison to men who reported ~13% [34]. Women in earlier career stages (e.g., postdoctoral fellowships or other early-career scientists) may have been most impacted by this childcare gap, as they are most likely to have young children [35]. Young children combined with the need for productivity during these formative career stages may have furthered gender gaps in academia. In British Columbia, Canada, mothers reported feeling like ‘bad moms’ in response to juggling household tasks and childcare [36].

IPV was heightened during COVID-19, with support services across Canada and within Ontario reporting an increased rate of IPV [37]. Men and women reported heightened concerns regarding physical and emotional abuse; in one case, men reported being more concerned about this than women surveyed [38]. Support resources for IPV were limited by stay-at-home orders, leaving providers to hide their own homes to provide confidential care [33], while IPV survivors struggled to access virtual appointments due to privacy concerns [39]. Patterns of violence against women and IPV were examined with Twitter data; women were identified as having experienced disproportionate violence [40]. IPV providers reported four main themes of IPV during COVID-19: no escape, isolation, complex decision making, and increased vulnerability [41]. These themes illustrate that IPV supports for women were less accessible.

### 1.5. Mental Health and COVID-19

Canadian adults’ average self-reported mental health score during COVID-19 was lower than the average prior to the pandemic [42]. Some adults in Canada reported an increase in junk food intake and no recreational physical activity [42], indicating that self-care behaviors may have declined during COVID-19. The proportion of all-cause emergency admissions attributed to alcohol increased during the first six months of COVID-19, although the proportion of alcohol-related admissions declined [20]. Hospital visits for acute alcohol poisoning decreased, and there was a decrease in visits for chronic alcohol use, although to a lesser extent, and in some instances, an increase [20]. In rural Ontario, alcohol-related emergency visit rates remained stable in comparison to pre-pandemic visits [20]. Non-physician-provided mental healthcare is not covered by the Ontario Health Insurance Plan (OHIP), leaving many without access to mental health support [43].

Mental health outcomes may be measured via subjective measures or functional health measures. Subjective health measures are measures based on individuals’ own perceptions of their own mental health [44]. Functional health measures evaluate how daily activities may be interrupted [45]. Perceived mental health measures, such as the one used in this analysis, have been found to be moderately correlated with poor physical health, increased healthcare utilization, and less satisfaction with mental health services [46]. Individuals who self-reported internalized poor mental health (e.g., depression and anxiety) have been shown to be experiencing similar symptoms to those already diagnosed with mental health conditions [47]. Further, perceived mental health may be a mediator between mental illness and well-being [48]. Patient-reported outcome measures (PROMs) are measures of patient perception of their health outcomes prior to and after a given intervention (cite). These measures have been shown to have significant clinical relevance, as in psychiatric settings PROMs have been linked to both the severity and progression of mental health outcomes [49]. PROMs have also been linked to improved clinical outcomes, specifically for depression [50]. It has been suggested that self-perceived mental health status may be a method used to triage support systems.

Measures of mental health during the COVID-19 pandemic were often based on validated scales or measures of self-perceived mental health; the absence of health professionals in responding to mental health scales may be linked to greater bias than when used in a clinical setting [51]. There are limited studies on mental healthcare utilization among Ontarians during the pandemic; however, acute mental health service access declined [52] while some sub-populations (birthing parents, physicians) reported increased utilization of virtual mental health supports [53,54]. Despite other research using similar subjective measures of mental health, this research is uniquely focused on rural populations and the drivers of added stress during the COVID-19 pandemic.

## 2. Materials and Methods

### 2.1. Study Area and Sample

Residents aged 18 or older living in Bruce (population density per square kilometer (pop/km^2^): 18.0, total population (pop): 73,396), Dufferin (pop/km^2^: 44.6, pop: 66,257), North Durham (only three municipalities: Scugog, Uxbridge, and Brock, pop: 55,704), Elgin (pop/km^2^: 50.4, pop: 94,752), Grey (pop/km^2^: 22.4, pop: 100,905), Middlesex (pop/km^2^: 150.9, pop: 78,239), and Oxford (pop/km^2^: 59.7, pop: 121,781) were included [55]. These counties are rural, with population densities of less than 400 people per square kilometer [56].

Surveys for these rural western Ontario counties were distributed via Canada Post and online. Data were collected from September 2021 to November 2021. Surveys were mailed to each known individual residence within the study areas. Survey respondents made up the survey sample.

### 2.2. Data Sources

#### 2.2.1. Survey Data

A cross-sectional survey was designed to establish topics relevant to rural experiences. The survey questions on health were based on the United Kingdom’s National Health Service (NHS) Health Survey for England [57]. An advisory board of six individuals working in local government, health units, non-profits, or service providers reviewed the proposed topics and provided feedback. Experts in the topic areas selected were asked to provide input. The survey was finalized with five main subjects of interest: demographics, individual well-being, social behaviour, mental health, and risk planning. A pilot was distributed to Perth and Huron Counties, and responses were collected between August and November 2020 [5].

After the pilot survey study, the survey was revised to include additional questions (e.g., childcare). Pilot work was presented at the Western Ontario Wardens Caucus, and counties were offered survey access. County selection within the caucus was limited by funding; therefore, the first seven counties to request the survey were selected for participation. The question on gender was asked ‘How do you describe your gender?’. The responses available were man, woman, non-binary, and ‘I use a different pronoun or prefer not to answer”.

The selected outcome of interest was the survey question ‘How would you rate your mental health?’ for both the before COVID-19 section of the analysis and after COVID-19. If a respondent selected poor mental health, they were coded as ‘cases’ or ‘1’. If a respondent selected excellent, good, average, or satisfactory, then they were coded as ‘controls’ or ‘0’. Non-response to this question resulted in exclusion. This measurement method has been used by Statistics Canada in the Canadian Community Health Survey, the Canadian Social Survey, and the Survey Series on People and their Communities [58]. The objective of using this measure was to evaluate overall self-perception of mental health during the pandemic.

#### 2.2.2. Census Data

Census data were collected from the University of Toronto Computing in the Social Sciences and Humanities (CHASS) Data Centre [55]. The 2021 Canadian census year was selected as it was the closest census year to the year of survey collection. While CHASS has variables on biological sex, the census’ newly introduced gender measures were not available via CHASS.

#### 2.2.3. COVID-19 Data

COVID-19 case data were collected through Ontario’s Case and Contact Management system (CCM) [59]. The data were limited to the start of COVID-19 until the end of the study period (November 2021) and then aggregated to a count of total cases per study county. Case rates were derived by dividing the number of total cases for the period by the county population and multiplying by 100,000.

### 2.3. Statistical Methods

#### 2.3.1. Data Cleaning and Summary Statistics

Data were aggregated into one Stata dataset per county and then analyzed with R-Statistical Programming Software (R) version 2024.04 (2024.04.0+735), ‘Puppy Cup’ [60]. Responses were compiled into one dataset for analysis. Survey participants who did not respond to the gender portion of the survey OR responded as a gender other than man or woman were excluded. There was not a large enough sample to explore inequities of those respondents identifying as genderqueer.

Survey sample alignment with the population was estimated by comparing the demographic measurements of the counties to census demographic measurements. The census data were compared at the county level, except for Durham County, which was estimated using the population totals from the three municipalities surveyed (Scugog, Uxbridge, and Brock). The survey sample was divided into a sample of men and a sample of women for comparison of survey responses, and differences in responses were measured via chi-squared tests. If estimates significantly differed, they were included in the final statistical models. These descriptive statistics can be found in Table 1. Instances where ‘not applicable’ were collected (e.g., demographics) were combined with missing, with the exception of Table 3.

#### 2.3.2. Data Visualization

Study areas, odds ratios for each county, and COVID-19 case rates for the survey period were visualized to identify spatial relationships in the data using ArcGIS Pro 3.0 and R. Shapefiles were obtained from Statistics Canada [61]. Maps were formatted in the projection Lambert Conformal Conic, the projection utilized by Statistics Canada [62]. Stacked bar charts were created to visualize the differing proportions of responses for men and women prior to and during COVID-19.

#### 2.3.3. Statistical Models

To evaluate differences in COVID-19 mental health by gender, unadjusted odds ratios were calculated for each county and overall using the formula OR = AD/BC, where A is exposure and event occurrence, B is exposure but no event occurrence, C is no exposure and event occurrence, and D is no exposure and no event occurrence. Exposure was the gender ‘woman’, and the event was self-report of ‘poor mental health’; men were considered the ‘unexposed’ group. Logistic regression models were used to produce adjusted odds ratios. Covariates were selected using the results found in Table 2; variables that significantly differed (*p*-value < 0.05) between men and women were included as a covariate, apart from housing situation and number of people in household. Housing situation was significantly associated with income (chi-squared test of independence, *p* < 0.05), and the number of people in a household was significantly associated with dependants in the home (chi-squared test of independence, *p*-value < 0.05).

Ethnicity, education, and primary income (i.e., employment) had greater than 5% of data missing; they were imputed using the RStudio Package ‘missMDA’, a package developed for imputing categorical data using Multifactor Correspondence Analysis (MCA) [63]. Children and other dependants were also included because they differed geographically.

#### 2.3.4. Spatial Approaches

Spatial analyses were used employed to evaluate variation in survey responses based on region. Building on the overall odds ratios and overall adjusted odds ratios, stratified odds ratios were calculated separately for each region. COVID-19 cases were then mapped on top of these odds ratios to examine if higher adjusted odds of poorer mental health were associated with higher case rates.

## 3. Results

### 3.1. Study Population

**Figure 1:**
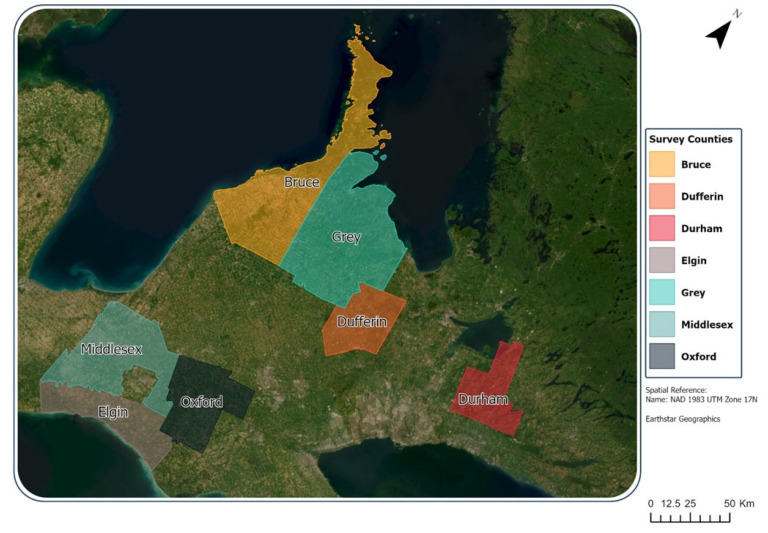
Map of study areas.

The study area (Figure 1) shows each county represented by a different color. The study area is spatially discontinuous, although most counties, except for Durham, share a border with at least one other county in the study. There were 18,864 survey respondents, and out of those, 11,978 reported their gender as woman, and 6211 reported their gender as man. These samples were sufficiently large and were retained in the study sample. In contrast, 32 respondents reported being non-binary, 54 indicated a preference not to disclose, and 589 respondents did not respond to the question.

The counties’ study sample does not completely align with the census county populations (Table 1). Gender identity was not available at the census division and subdivision level [55], so the proportions of gender were compared to sex. The census sample shows there are more females (50.6%) than males (49.4%) in the counties studied. The survey sample is unbalanced, with most respondents identifying as women (65.9%) and only (34.1%) identifying as men. The age distribution of the sample varied geographically, with Elgin County having the highest percentage of (75.3%) adults less than 70 and Grey County having the highest percentage of (36.6%) adults aged 70 or more. In contrast, the census sample’s highest percentage of adults less than 70 was in Elgin County (85.5%), and the highest percentage of adults aged greater than 70 was in North Durham (28.6%). The age distribution (Table 1) of the sample is older (29% being older than 70) than the census (20.1% of the population being over 70).

Visible minorities were slightly overrepresented in this sample (the proportion who identified as a visible minority was 8.0%) in comparison to the census (those who identified as a visible minority were 6.3%). Approximately 20% of the survey sample did not respond to this question. Educational attainment distributions differed between the census and the sample. In the survey sample, over 70% of respondents had obtained a bachelor’s degree or higher; only 46.3% of the census population had achieved this.

### 3.2. Survey Responses

The survey questions are stratified by gender identity in Table 2. The age distribution between men and women significantly differed. Men in the survey sample were older (aged 80 or more 10.4%) than women (aged 80 or more 6.0%), and women had higher proportions of respondents aged 59 or less for each age grouping in comparison to men. Education significantly differed, with a larger proportion of women obtaining a bachelor’s degree (58.9%) in comparison to men (45.4%); men in this sample (13.7%) had a larger percentage of graduate degrees than women (12%). More men had less than or equal to a high school degree (23.1% vs. women 22%). Men obtained a trades certificate (11.2%) more than women (3.3%). In terms of employment, more women reported being employed part-time (men: 2.4%, women: 7.5%), while more men reported not being in the workforce (men: 51.6%, women: 42.7%). About a fifth (~20%) of the sample’s ethnicity was missing for both men and women in the survey. The majority of those who did respond to the question were white (men: 75.2%, women: 72.6%).

Most survey respondents had lived in their respective communities for three or more years (men: 86.2%, women: 85.5%). For those who did report moving, men and women did not report significant differences in securing housing or in where they were moving from. Most men and women in the survey sample owned their homes (men: 88%, women: 85.1%). Women rented (10.7%) more often than men (8.7%); women (3.5%) were less likely to respond to this question than men (2.6%). Women in the sample had more individuals living in their home, with higher percentages for three people (men: 10.6%, women: 13.9%), four people (men: 8.3%, women: 11.8%), and more than four people (men: 5.2%, women: 6.9%). Women in the survey sample reported having more children or dependants in the home (men: 22.3%, women: 30.5%). Women reported accessing daycare services more and reported increased difficulty during COVID-19.

Self-reported mental health for all survey counties and regions was examined to establish if women reported different mental health status than men (Table 3). Prior to COVID-19, a larger percentage of men self-reported ‘excellent’ mental health in comparison to women (men: 30.5%, women: 24.4%). In contrast, women self-reported their mental health as ‘average’ more than men prior to COVID-19 (men: 10.8%, women 14.9%); neither had a high proportion of ‘poor’ self-reported mental health (men: 1.3%, women: 1.8%). After the start of COVID-19, fewer women reported ‘excellent’ mental health than before COVID-19 (men: 19.1%, women: 10.3%). In comparison to before the COVID-19 pandemic, more women and men reported ‘average’ mental health (men: 17.8%, women: 23.6%). The self-reported ‘poor’ mental health was greater in women and men after the start of COVID-19 (men: 6.6%, women: 13.2%).

The unadjusted (Table 4) odds ratios for poor mental health between men and women pre-pandemic were 1.34 with a 95% confidence interval between 1.04 and 1.75. The overall odds ratios for poor mental health between men and women post-pandemic were 2.12 with a 95% confidence interval between 1.89 and 2.38. The odds of a woman reporting ‘poor mental health’ pre-pandemic were 1.34 times that of men; the odds of a woman reporting ‘poor mental health’ during COVID-19 jumped to 2.12 times that of a man.

Overall pre-pandemic adjusted odds ratios (Table 5) were 1.09. At the 0.05 level, this odds ratio is not significant and indicates that self-reported poor mental health was the same for both men and women pre-pandemic. Mid-pandemic self-reported mental health was significant, with an odds ratio of 1.77, meaning the adjusted odds of a woman reporting poor mental health during COVID-19 were 1.77 times that of a man. Odds ratios with confidence intervals that do not cross one are considered significant.

**Figure 2:**
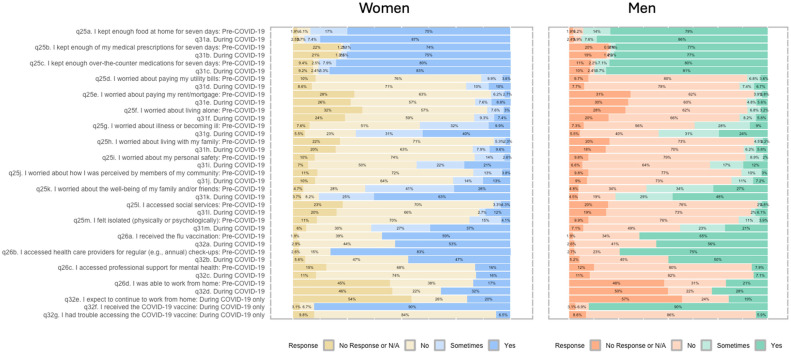
Men vs. women’s responses to mental health survey questions.

The segmented bar graph (Figure 2) demonstrates that women in the survey sample reported less support and more heightened worry during COVID-19 than men. Specifically, women were more worried about paying their utility bills (10% responded ‘yes’ when asked if worried) in comparison to men during the pandemic (7% responded ‘yes’ when asked if worried about rent). Similarly, during the pandemic, women were more worried about paying rent (9% women, 6% men; before COVID-19, 2% of men and 3% of women reported worry about paying rent), and women were far more worried about becoming ill during the pandemic than men (40% women, 24% men). Despite the increase in reported worry during the pandemic, mental health support remained stable for women (with 16% seeking a professional) and decreased slightly for men (pre-pandemic, 8% sought out professional help, and during the pandemic, 7% sought out professional help).

Health behaviors such as social interaction and substance consumption (Table 6) were evaluated to determine if they impacted the deterioration of mental health during COVID-19.

**Figure 3:**
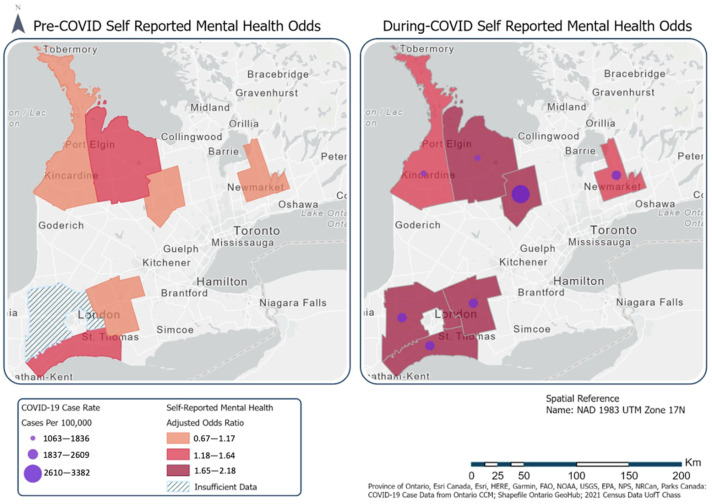
Map of self-reported mental health by county with COVID-19 case overlay.

In general, men reported using substances more than women; however, both genders reported an increase in substance or alcohol use during the pandemic in addition to a decline in spending time with friends or family. The final map (Figure 3) shows where self-reported mental was worst and is overlaid with the overall highest COVID-19 case rates for the survey time period.

### 3.3. Key Findings

The key findings of this analysis are that overall, women had a more dramatic shift in self-reported mental health and worse self-reported mental health than men. This pattern remained consistent after adjusting for possible imbalances in the data.

## 4. Discussion

This survey evaluated the impacts of COVID-19 on rural southern Ontario communities. The survey sample was more women (65.9%) than men (34.1%), which may indicate a response bias as the census reports only slightly more females (50.6%) living in the sampled counties. The differential response by gender is not uncommon, though, as women or females are more likely to respond to surveys than men or males. Gender response bias is a documented issue with mail, telephone, and internet surveys [64,65,66,67]. In surveys that are gender-inclusive, women are more likely to respond than men; similarly, in gender-specific surveys, women have a higher overall response rate than men [65]. In future surveys, incentivizing responses, providing reminders, or offering additional survey modes may improve survey responses [68]. Despite being outside of the scope of this analysis, propensity score matching is one proposed method for created a subset of matched pairs for analysis; this may better account for gender imbalances in the survey sample [69].

The census is not an ideal comparator. The survey measured gender, whereas the census measured sex. The survey did not collect data on gender versus biological sex, and therefore non-cisgender individuals were not captured, introducing opportunity for bias. Municipalities in North Durham County had the greatest gender disparity, with 69.9% of respondents self-reporting as women and only 30.1% of respondents self-reporting as men. Respondents identifying as women had a younger age distribution than men. Women reported having a higher percentage of bachelor’s degrees, while men in the sample had a higher percentage of graduate-level degrees; education, however, influences response bias, and this may be a reflection of that, especially among the men [70]. Women in the sample appeared to fill part-time roles more than full-time roles, while men in the sample had a higher percentage of full-time employment. These results align with research on gender equity in employment during COVID-19, which showed that women were more often unemployed. This work indicated that advancement of women in the workplace during COVID-19 slowed as a result of household and childcare responsibilities in the home [71].

Self-reported mental health for both men and women worsened from pre-COVID-19 to during COVID-19. Almost twice the percentage of women self-reported ‘poor’ mental health during COVID-19 in comparison to men. A Canada-wide survey had similar findings; males had less self-reported worsened mental health than females during COVID-19 [72]. A scoping review found women had higher odds of reporting poor mental health conditions in comparison to men, as did people living in rural areas in comparison to urban areas [73]. When odds ratios were adjusted for age, education, primary source of economic support, and number of dependants, the odds of women reporting poor mental health during COVID-19 remained almost twice that of men. The odds of women reporting poor mental health during COVID-19 differed by county, indicating spatial variation even within rural areas. This aligns with similar findings in the United States, where there was variation in mental health outcomes between rural and semi-rural communities [74]. Rural areas each have unique social determinants of mental health outcomes [75]. In the context of mental health intervention, some rural areas may have poorer access to mental healthcare, and alternative interventions may become more important as a result [76]. The geographies of alternative mental health interventions (e.g., greenspaces) are not spatially homogenous and may differentially impact mental health [77].

The survey herein evaluated factors which may have contributed to poorer mental health—stressors and health behaviors. Stressors (Figure 2) were evaluated by asking respondents to gauge their level of worry pre and during COVID-19. The during-pandemic responses to ‘I worried about my personal safety’ increased for both men and women respondents, with 21% of women responding ‘Yes’ and 22% responding ‘Sometimes’; some 12% of men responded ‘Yes’, and 17% of men responded ‘Sometimes’. This may be a reflection of increased intimate partner violence that occurred during COVID-19 [78]. In rural settings, intimate partner violence interventions such as in-home support [79] as well as facilitated group discussions [80] can be effective; these were not possible during lockdowns. These interventions rely upon social support, which became another stressor for women in the sample during COVID-19. The responses to the survey question ‘I felt isolated physically or psychologically’ pre-pandemic were 4% ‘Yes’ for women and 4% for men. During the pandemic, 37% of women responded ‘Yes’, and 21% of men responded ‘Yes’; men may have felt isolated, but in this sample, not to the same extent as women. Social supports have been linked to mental health; female university students who had greater social support had reduced anxiety, depression, and stress symptoms [81]. Social supports may have a greater impact on female mental health symptoms in comparison to males [82]; these findings show that males may be more resilient to isolation [81,82]. Despite experiences of decreased perceived safety and increased isolation, reported mental health resource access did not differ for women during COVID-19, and increased only 1% from before to during the pandemic in men, possibly indicating that there are barriers to or stigma associated with accessing mental health support. Few sampled women and men reported seeing their family members never or not at all prior to COVID-19; during the pandemic, however, 19% of women and 14% of men reported no family contact.

Similar to this study, women living in rural communities in Manitoba and internationally described isolation and a loss of autonomy as a result of COVID-19 [29,83]. Resources for women living in rural environments during the pandemic were limited [84,85]. Social support and social belonging are key to good mental health [27]; rural communities faced far more isolation and barriers to accessing community [85]. Transportation, childcare, and autonomy to leave unsafe environments have been noted as areas in which women and children living in rural environments need more support [85]. In addition to removing social supports and addition of stressors, women may internalize stress more than men, with studies indicating women experience more depression and anxiety after stress exposure [86,87].

Reported alcohol and marijuana use increased for men and women during COVID-19, indicating substance use as a coping mechanism [88]. During COVID-19, 21.5% of women reported consuming alcohol more than twice a week, and 5.2% reported consuming marijuana more than twice a week. The increase in consumption of alcohol by women may be in response to increased stress [89,90]. While men reported increased use, the magnitude of increase for both substances was larger for women respondents. Men are more likely to seek out alcohol following stress [87]. Out of all of the provinces, Ontario had the largest increase (30%) in alcohol consumption rates [91]. The top three reasons for the increase in alcohol consumption in Canada were boredom, stress, and convenience [91]. The reports of an increase in drinking accounted for less than 50% of individuals who reported consuming alcohol [91], but within this subgroup, there may have been more people at risk of alcohol dependence as a result of time at home, boredom, and access (liquor stores were an emergency service [92]). COVID-19 compounded many risk factors of alcohol dependence [93]. Mortality in Canada fully attributed to alcohol sharply increased (by over 17%) from April 2020 to December 2022 [94]. Alcohol abuse may have disproportionately impacted rural environments, which has been demonstrated by increased alcohol-related emergency department visits [95].

The Ontario 2024 report from the domestic violence death review committee revealed that alcohol was involved in 41% of domestic violence deaths [96]. In the years following 2020, the rate of police-reported intimate partner violence (2020—242 per 100,000 people, 2021—249 per 100,000 people, 2022—257 per 100,000 people) and family violence (2020—204 per 100,000 people, 2021—214 per 100,000 people, 2022—221 per 100,000 people) has increased as well [97]. These rates coupled with the increases in substance use in the study sample may indicate that higher substance use could be associated with more instances of IPV, especially in rural communities. Alcohol and substance use should be a consideration in future interventions. Public resources for IPV and substance dependence should be well understood and available via Internet and telephone (for those too remote for Internet connection).

The final figure shows counties with high cumulative COVID-19 case rates may have higher adjusted odds of poor mental health. This possibility could be examined in future work, as it may be valuable to understand how mental health changed in response to the severity of COVID-19 in rural communities.

Survey-based studies of rural communities during COVID-19 were widespread internationally. Gender and mental health focuses were rarer, however. An Australian study examined the relationship between rural populations in first nations and non-first nations and determined that rural first-nations populations were more worried about the harmfulness of COVID-19 and its economic impacts [98]. This supports the idea that socioeconomically disadvantaged groups (e.g., first nations and women) experienced heightened worry because of financial stressors. A nationwide study in the US found that food insecurity was also heightened in rural counties during the pandemic, despite efforts to prevent it [99]. This bolsters the idea that women had additional financial stressors within the study sample. Food insecurity is both a rural and women’s issue, disproportionately impacting women [100] and those who live in rural areas [101]. While most women and men in this research study reported having enough food at home, efforts to ensure this may have led to additional financial strain.

In addition, social cohesion was a notable source of support in the study population. A rural Alabama study suggests that social networks and community figures (e.g., religious leaders) influenced vaccine uptake [102]. These networks in rural communities should be leveraged for health communication. If people are receiving health information from their peers, information may be more effectively disseminated through community organizations or priests.

### Limitations

This sample is not representative of the general populations living in the seven counties surveyed. This sample was older, more educated, and had more visible minorities than the census. Non-binary and other gender identities were not well captured by this survey; in the analysis process, it was impossible to discern the difference between individuals who utilized other pronouns versus those who preferred not to answer. As a result of this, genders other than women and men were not included. This is a major limitation, as gender exists on a spectrum and there are many more gender identities and expressions than man and woman [103]. Gender, sex, and sexuality may often be confused [104]; future surveys present an opportunity for public education on the subject while ascertaining more information on the non-binary and queer populations in rural areas.

This survey did not collect biological sex data or data on sexuality, so it is unknown if the respondents in the home were living in heteronormative households; these are important considerations in understanding how social support may impact mental health outcomes [105]. There is a need for additional research that includes non-gender-conforming identities [106]. A Canada-wide analysis indicates that trans and non-binary individuals faced increased financial stressors, strained social networks, and additional worry about safety. These findings are similar to those of women in the study population. Excluding gender minorities means that there may be social groups that suffered and are not acknowledged.

The sample could have been improved by expanding to include urban and suburban counties as comparators. In a European study, women in urban environments were more likely to report poorer perceived mental health; however, women with children had higher odds of reporting poorer mental health, and women living in rural environments perceived poor mental health in the context of a perceived increase in violence against women [107]. Within Europe, there remained differences in perceived mental health across rural areas [107], demonstrating again that rural communities are unique and need region-specific interventions. To further illustrate this point, future work should explore rural communities in other provinces as well.

## 5. Conclusions

Women surveyed in this study reported increased isolation and greater concern for their personal safety. Respondents who self-identified as women were slightly less educated, younger, and had more dependants than those who self-identified as men. Women reported poor mental health during COVID-19 more than men, even with covariate adjustments. Odds ratios differed across the counties surveyed for this study, underscoring the importance of accounting for spatial variation within rural areas. Women and men reported increased substance use during COVID-19. There is a strong need for rural [108] and gender-specific interventions during pandemics or other distressing events. Women may experience increased burden of household work; it may become impossible to balance career, childcare, and household work when the demands for each increase. Women need social support to thrive; an increase in social isolation will reduce women’s’ protections from poor mental health outcomes. It is imperative that we take steps to ensure supports for women during future pandemics; gender-based health equity must be prioritized in hazard planning.

## Figures and Tables

**Table 1:** Sample comparison (gender, age, visible minority, and education) ^a^.

	Bruce		Dufferin		Elgin		Grey		Middlesex ^b^		North Durham ^c^		Oxford		Overall	
	Sample N: 2459 N (%)	Census N: 73,396 N (%)	Sample N: 2157 N (%)	Census N: 66,257 N (%)	Sample N: 2150 N (%)	Census N: 94,752 N (%)	Sample N: 3763 N (%)	Census N: 100,905 N (%)	Sample N: 2779 N (%)	Census N: 78,239 N (%)	Sample N: 1663 N (%)	Census N: 55,704 N (%)	Sample N: 3218 N (%)	Census N: 121,781 N (%)	Sample N: 18,189 N (%)	Census N: 591,034 N (%)
Gender/Sex ^d^																
Man	896 (36.4%)		698 (32.4%)		712 (33.1%)		1312 (34.9%)		958 (34.5%)		525 (31.6%)		1110 (34.5%)		6211 (34.1%)	
Woman	1563 (63.6%)		1459 (67.6%)		1438 (66.9%)		2451 (65.1%)		1821 (65.5%)		1138 (68.4%)		2108 (65.5%)		11,978 (65.9%)	
Male		36,355 (49.5%)		32,775 (49.5%)		46,735 (49.3%)		49,770 (49.3%)		38,930 (49.8%)		27,485 (49.3%)		60,155 (49.4%)		292,205 (49.4%)
Female		37,040 (50.5%)		33,480 (50.5%)		48,020 (50.7%)		51,135 (50.7%)		39,305 (50.2%)		28,225 (50.7%)		61,625 (50.6%)		298,830 (50.6%)
Age																
18–69 ^e^	1627 (66.2%)	45,280 (78.1%)	1591 (73.8%)	42,705 (85.5%)	1618 (75.3%)	58,800 (81.6%)	2351 (62.5%)	62,160 (77.2%)	2037 (73.3%)	48,750 (82.0%)	1182 (71.1%)	31,520 (71.4%)	2377 (73.9%)	75,915 (81.7%)	12,783 (70.3%)	642,130 (79.9%)
70+	815 (33.1%)	12,730 (21.9%)	550 (25.5%)	7270 (14.5%)	518 (24.1%)	13,290 (18.4%)	1379 (36.6%)	18,390 (12.8%)	729 (26.2%)	10,710 (18.0%)	472 (28.4%)	12,610 (28.6%)	813 (25.3%)	16,970 (18.3%)	5276 (29.0%)	144,350 (20.1%)
Visible Minority Status																
Not Visible Minority (i.e., white)	1743 (70.9%)	69,355 (96.2%)	1573 (72.9%)	55,095 (84.1%)	1583 (73.6%)	89,145 (95.4%)	2709 (72.0%)	94,985 (95.8%)	2064 (74.3%)	74,535 (96.6%)	1284 (77.2%)	51,590 (93.8%)	2408 (74.8%)	111,035 (92.3%)	13,364 (73.5%)	545,740 (93.7%)
Visible Minority	198 (8.1%)	2745 (3.8%)	209 (9.7%)	10,390 (15.9%)	194 (9.0%)	4275 (4.6%)	283 (7.5%)	4175 (4.2%)	208 (7.5%)	2655 (3.4%)	124 (7.5%)	3405 (6.2%)	240 (7.5%)	9230 (7.7%)	1456 (8.0%)	36,875 (6.3%)
Highest Educational Attainment																
High School or Less	560 (22.8%)	27,050 (45.1%)	445 (20.6%)	25,800 (48.5%)	465 (21.6%)	40,155 (52.6%)	941 (25.0%)	41,735 (49.8%)	553 (19.9%)	27,770 (44.0%)	330 (19.8%)	21,280 (45.7%)	2408 (74.8%)	51,975 (52.9%)	4062 (22.3%)	235,765 (49.0%)
Undergraduate Degree or Some College	1477 (60.1%)	30,020 (50.0%)	1284 (59.5%)	24,675 (46.3%)	1369 (63.7%)	33,555 (43.9%)	2143 (56.9%)	37,450 (44.7%)	1756 (63.2%)	31,645 (50.2%)	1006 (60.5%)	22,670 (48.7%)	1942 (60.3%)	42,780 (43.5%)	10,977 (60.3%)	222,795 (46.3%)
Graduate Degree or Certificate	292 (11.9%)	2970 (4.9%)	319 (14.8%)	2775 (5.2%)	215 (10.0%)	2690 (3.5%)	497 (13.2%)	4580 (5.47)	351 (12.6%)	3655 (5.8%)	259 (15.6%)	2640 (5.7%)	351 (10.9%)	3505 (3.6%)	1787 (9.8%)	22,815 (4.7%)

^a^ Missing data are supressed from table, so totals may not add up to 100%. ^b^ Middlesex does not include London, Ontario (an urban centre). ^c^ North Durham includes only rural cities within North Durham (i.e., Scugog, Uxbridge, and Brock). ^d^ Survey collected gender identity, but census collected sex. ^e^ Age data collected by survey are 18+, age data collected by the census go from 20+.

**Table 2:** Men vs. women survey demographics.

	Men (N = 6211)	Women (N = 11,978)	*p*-Value
How old are you?			
18–29 years	184 (3.0%)	583 (4.9%)	<0.001
30–39 years	486 (7.8%)	1475 (12.3%)	
40–49 years	527 (8.5%)	1583 (13.2%)	
50–59 years	876 (14.1%)	2243 (18.7%)	
60–69 years	1740 (28.0%)	3086 (25.8%)	
70–79 years	1712 (27.6%)	2197 (18.3%)	
80+ years	647 (10.4%)	720 (6.0%)	
Missing	39 (0.6%)	91 (0.8%)	
What is your highest level of education completion?			
Grade 13 or less	1449 (23.4%)	2646 (22.1%)	<0.001
Trades certificate	697 (11.2%)	400 (3.3%)	
Undergraduate degree/College diploma	2822 (45.4%)	7058 (58.9%)	
Post-graduate degree (e.g., Master’s, PhD, MD)	850 (13.7%)	1434 (12%)	
Missing	393 (6.3%)	440 (3.7%)	
Which of the following best describes your primary source of economic support?			
Unemployed	75 (1.2%)	312 (2.6%)	<0.001
Employed Part-Time	148 (2.4%)	899 (7.5%)	
Employed Full-Time or Self-Employed	2550 (41.1%)	5027 (42.0%)	
Not in Work Force (Student, Social Assistance or Retired)	3204 (51.6%)	5117 (42.7%)	
Missing	234 (3.8%)	623 (5.2%)	
Do you identify with any of the ethnicities listed below?			
Asian Identity	95 (1.5%)	105 (0.9%)	0.001
Black Identity	16 (0.3%)	50 (0.4%)	
Indigenous Identity	48 (0.8%)	105 (0.9%)	
White Identity	4672 (75.2%)	8692 (72.6%)	
Other Identity	359 (5.8%)	678 (5.7%)	
Missing	1021 (16.4%)	2348 (19.6%)	
Have you lived in the community for less than three years?			
Yes	806 (13.0%)	1616 (13.5%)	0.326
No	5352 (86.2%)	10,243 (85.5%)	
Missing	53 (0.9%)	119 (1.0%)	
Did you experience trouble securing housing when you moved to the area?			
Yes	130 (2.1%)	309 (2.6%)	0.067
No	690 (11.1%)	1321 (11.0%)	
Missing	5391 (86.8%)	10,348 (86.4%)	
Where did you move from?			
Greater Toronto Region (GTA)	241 (3.9%)	453 (3.8%)	0.895
Elsewhere in Ontario (excluding GTA)	487 (7.8%)	1010 (8.4%)	
Within Canada (excluding Ontario)	32 (0.5%)	67 (0.6%)	
The United States	5 (0.1%)	11 (0.1%)	
Elsewhere	21 (0.3%)	43 (0.4%)	
Missing	5425 (87.3%)	10,394 (86.8%)	
Which of the following best describes your housing situation?			
Own	5464 (88.0%)	10,195 (85.1%)	<0.001
Rent	541 (8.7%)	1283 (10.7%)	
Retirement or Long-Term Care	14 (0.2%)	22 (0.2%)	
Other	28 (0.5%)	55 (0.5%)	
Missing	164 (2.6%)	423 (3.5%)	
In addition to yourself, how many people currently live in your home?			
0	308 (5.0%)	546 (4.6%)	<0.001
1	1572 (25.3%)	2722 (22.7%)	
2	2769 (44.6%)	4679 (39.1%)	
3	657 (10.6%)	1667 (13.9%)	
4	518 (8.3%)	1408 (11.8%)	
More than four	321 (5.2%)	823 (6.9%)	
Missing	66 (1.1%)	133 (1.1%)	
Do you have children or dependants at home?			
No	4749 (76.5%)	8167 (68.2%)	<0.001
Yes	1388 (22.3%)	3655 (30.5%)	
Missing	74 (1.2%)	156 (1.3%)	
Do you access childcare services (e.g., daycare)?			
No	1123 (18.1%)	2861 (23.9%)	0.142
Yes	262 (4.2%)	753 (6.3%)	
Missing	4826 (77.7%)	8364 (69.8%)	
Have you experienced difficulty securing daycare services?			
Yes	99 (1.6%)	394 (3.3%)	<0.001
No	173 (2.8%)	382 (3.2%)	
Missing	5939 (95.6%)	11,202 (93.5%)	
Location			
Bruce	896 (14.4%)	1563 (13.0%)	0.014
Dufferin	698 (11.2%)	1459 (12.2%)	
Elgin	712 (11.5%)	1438 (12.0%)	
Grey	1312 (21.1%)	2451 (20.5%)	
Middlesex *	958 (15.4%)	1821 (15.2%)	
North Durham ^+^	525 (8.5%)	1138 (9.5%)	
Oxford	1110 (17.9%)	2108 (17.6%)	

* Middlesex does not include London, Ontario (an urban centre). ^+^ North Durham includes only rural cities within North Durham (i.e., Scugog, Uxbridge, and Brock).

**Table 3:** Survey sample’s mental health in rural counties.

Prior to COVID-19				Since the Start of COVID-19 (After 1 March 2020)		
	Man (N = 6211)	Woman (N = 11,978)	*p*-Value	Man (N = 6211)	Woman (N = 11,978)	*p*-Value
How would you rate your mental health?						
Excellent	1892 (30.5%)	2928 (24.4%)	<0.001	1185 (19.1%)	1238 (10.3%)	<0.001
Good	3168 (51.0%)	6140 (51.3%)		2660 (42.8%)	4014 (33.5%)	
Average	672 (10.8%)	1780 (14.9%)		1105 (17.8%)	2826 (23.6%)	
Satisfactory	279 (4.5%)	740 (6.2%)		697 (11.2%)	2088 (17.4%)	
Poor	82 (1.3%)	213 (1.8%)		409 (6.6%)	1562 (13.0%)	
Not applicable	10 (0.2%)	11 (0.1%)		35 (0.6%)	54 (0.5%)	
Missing	108 (1.7%)	166 (1.4%)		120 (1.9%)	196 (1.6%)	

**Table 4:** Survey sample unadjusted odds ratios of mental health stratified by self-reported gender.

Overall (All Surveyed Counties)							
Pre-Pandemic Odds Ratios				Mid-Pandemic Odds Ratio			
	Poor Mental Health		Not Poor Mental Health		Poor Mental Health		Not Poor Mental Health
Woman	208		11,424	Woman	1551		10,047
Man	80		5982	Man	405		5555
OR (95% CI)		1.34 (1.04, 1.75)		OR (95% CI)		2.12 (1.89, 2.38)	
Bruce County							
	Poor Mental Health		Not Poor Mental Health		Poor Mental Health		Not Poor Mental Health
Woman	21		1497	Woman	139		1372
Man	14		840	Man	42		810
OR (95% CI)		0.84 (0.43, 1.70)		OR (95% CI)		1.95 (1.38, 2.81)	
Dufferin County							
	Poor Mental Health		Not Poor Mental Health		Poor Mental Health		Not Poor Mental Health
Woman	18		1403	Woman	179		1238
Man	11		655	Man	39		623
OR (95% CI)		0.76 (0.36, 1.68)		OR (95% CI)		2.30 (1.62, 3.34)	
Elgin County							
	Poor Mental Health		Not Poor Mental Health		Poor Mental Health		Not Poor Mental Health
Woman	36		1365	Woman	233		1165
Man	10		679	Man	62		624
OR (95% CI)		1.77 (0.90, 3.81)		OR (95% CI)		2.01 (1.50, 2.72)	
Grey County							
	Poor Mental Health		Not Poor Mental Health		Poor Mental Health		Not Poor Mental Health
Woman	47		2319	Woman	256		2100
Man	12		1242	Man	70		1181
OR (95% CI)		2.08 (1.13, 4.13)		OR (95% CI)		2.05 (1.57, 2.72)	
Middlesex County							
	Poor Mental Health		Not Poor Mental Health		Poor Mental Health		Not Poor Mental Health
Woman	26		1750	Woman	256		1517
Man	***		927	Man	63		866
OR (95% CI) ***		N/A		OR (95% CI)		2.31 (1.75, 3.11)	
North Durham (Partially Durham County)							
	Poor Mental Health		Not Poor Mental Health		Poor Mental Health		Not Poor Mental Health
Woman	19		1088	Woman	152		953
Man	6		501	Man	40		466
OR (95% CI)		1.43 (0.60, 4.01)		OR (95% CI)		1.85 (1.30, 2.70)	
Oxford County							
	Poor Mental Health		Not Poor Mental Health		Poor Mental Health		Not Poor Mental Health
Woman	41		2002	Woman	336		1702
Man	23		1058	Man	89		985
OR (95% CI)		0.94 (0.56, 1.60)		OR (95% CI)		2.18 (1.71, 2.81)	

*** Odds ratios were not computed for cell counts less than 5.

**Table 5:** Adjusted odds ratios.

Overall (All Surveyed Counties)					
Pre-Pandemic Odds Ratios			Mid-Pandemic Odds Ratio		
	Poor Mental Health	Not Poor Mental Health		Poor Mental Health	Not Poor Mental Health
Adjusted OR (95% CI)	1.09 (0.84, 1.43)		Adjusted OR (95% CI)	1.77 (1.58, 2.00)	
Bruce County					
	Poor Mental Health	Not Poor Mental Health		Poor Mental Health	Not Poor Mental Health
Adjusted OR (95% CI)	0.74 (0.37, 1.52)		Adjusted OR (95% CI)	1.55 (1.09, 2.27)	
Dufferin County					
	Poor Mental Health	Not Poor Mental Health		Poor Mental Health	Not Poor Mental Health
Adjusted OR (95% CI)	0.67 (0.31, 1.53)		Adjusted OR (95% CI)	1.96 (1.37, 2.87)	
Elgin County					
	Poor Mental Health	Not Poor Mental Health		Poor Mental Health	Not Poor Mental Health
Adjusted OR (95% CI)	1.48 (0.74, 3.25)		Adjusted OR (95% CI)	1.78 (1.31, 2.44)	
Grey County					
	Poor Mental Health	Not Poor Mental Health		Poor Mental Health	Not Poor Mental Health
Adjusted OR (95% CI)	1.63 (0.88, 3.26)		Adjusted OR (95% CI)	1.69 (1.27, 2.26)	
Middlesex County					
	Poor Mental Health	Not Poor Mental Health		Poor Mental Health	Not Poor Mental Health
Adjusted OR (95% CI)	N/A***		Adjusted OR (95% CI)	2.18 (1.62, 2.96)	
North Durham (Partial Durham County)					
	Poor Mental Health	Not Poor Mental Health		Poor Mental Health	Not Poor Mental Health
Adjusted OR (95% CI)	1.01 (0.40, 2.93)		Adjusted OR (95% CI)	1.40 (0.96, 2.07)	
Oxford County					
	Poor Mental Health	Not Poor Mental Health		Poor Mental Health	Not Poor Mental Health
Adjusted OR (95% CI)	0.77 (0.46, 1.34)		Adjusted OR (95% CI)	1.93 (1.50, 2.50)	

*** Odds ratios were not computed for cell counts less than 5.

**Table 6:** Health behaviors.

Prior to COVID-19				Since the Start of COVID-19 (After 1 March 2020)		
	Men (N = 6211)	Women (N = 11,978)	*p*-Value	Man (N = 6211)	Woman (N = 11,978)	*p*-Value
How often did you spend time with friends and/or family?						
More than 2x/week	2387 (38.4%)	5380 (44.9%)	<0.001	739 (11.9%)	998 (8.3%)	<0.001
1–2 times/week	2351 (37.9%)	4306 (35.9%)		1326 (21.3%)	2139 (17.9%)	
1–2x/month	1297 (20.9%)	2035 (17.0%)		3058 (49.2%)	6075 (50.7%)	
Never or not at all	58 (0.9%)	73 (0.6%)		870 (14.0%)	2280 (19.0%)	
Missing	118 (1.9%)	184 (1.5%)		218 (3.5%)	486 (4.1%)	
How often did you consume alcohol?						
More than 2x/week	1787 (28.8%)	1886 (15.7%)	<0.001	1975 (31.8%)	2572 (21.5%)	<0.001
1–2 times/week	1626 (26.2%)	2893 (24.2%)		1403 (22.6%)	2422 (20.2%)	
1–2x/month	1381 (22.2%)	3524 (29.4%)		1189 (19.1%)	2730 (22.8%)	
Never or not at all	1282 (20.6%)	3450 (28.8%)		1461 (23.5%)	3850 (32.1%)	
Missing	135 (2.2%)	225 (1.9%)		183 (2.9%)	404 (3.4%)	
How often did you consume marijuana?						
More than 2x/week	313 (5.0%)	445 (3.7%)	<0.001	391 (6.3%)	623 (5.2%)	0.008
1–2 times/week	166 (2.7%)	244 (2.0%)		182 (2.9%)	311 (2.6%)	
1–2x/month	293 (4.7%)	570 (4.8%)		299 (4.8%)	575 (4.8%)	
Never or not at all	5329 (85.8%)	10,546 (88.0%)		5177 (83.4%)	10,177 (85.0%)	
Missing	110 (1.8%)	173 (1.4%)		162 (2.6%)	292 (2.4%)	
How often did you consume opioids?						
More than 2x/week	33 (0.5%)	75 (0.6%)	0.293	39 (0.6%)	75 (0.6%)	0.887
1–2 times/week	12 (0.2%)	15 (0.1%)		14 (0.2%)	24 (0.2%)	
1–2x/month	17 (0.3%)	48 (0.4%)		22 (0.4%)	51 (0.4%)	
Never or not at all	6050 (97.4%)	11,680 (97.5%)		5972 (96.2%)	11,532 (96.3%)	
Missing	99 (1.6%)	160 (1.3%)		164 (2.6%)	296 (2.5%)	

## Data Availability

Survey data is confidential and is only accessible for those listed on the IRB or at aggregate levels where survey participants cannot be identified. If there are questions regarding the analysis or the data, the authors are happy to answer them via email.
